# Protective Effects of Red Guava on Inflammation and Oxidative Stress in Streptozotocin-Induced Diabetic Mice

**DOI:** 10.3390/molecules201219831

**Published:** 2015-12-12

**Authors:** Pei-Ying Li, Cheng-Chin Hsu, Mei-Chin Yin, Yueh-Hsiung Kuo, Feng-Yao Tang, Che-Yi Chao

**Affiliations:** 1 Department of Health and Nutrition Biotechnology, Asia University, Taichung 41354, Taiwan; chacolate042276@yahoo.com.tw; 2 School of Pharmacy, College of Pharmacy, China Medical University, Taichung 40402, Taiwan; 3 Department of Nutrition, Chung Shan Medical University, Taichung, 40201, Taiwan; king@csmu.edu.tw; 4 Department of Nutrition, China Medical University, Taichung 40402, Taiwan; mcyin@mail.cmu.edu.tw (M.-C.Y.); vincenttang@mail.cmu.edu.tw (F.-Y.T.); 5 Department of Biotechnology, Asia University, Taichung 41354, Taiwan; kuoyh@mail.cmu.edu.tw; 6 Department of Chinese Pharmaceutical Sciences and Chinese Medicine Resources, China Medical University, Taichung 40402, Taiwan; 7 Department of Medical Research, China Medical University Hospital, China Medical University, Taichung 40447, Taiwan

**Keywords:** red guava, diabetes, anti-inflammation, oxidative stress

## Abstract

Diabetes is an important chronic disease and the 4th leading cause of death in Taiwan. Hyperglycemia-induced oxidative and inflammatory damage are the main causes of chronic complications in diabetic patients. The red guava (red-fleshed guava cultivar of *Psidium guajava* L.) is a tropical fruit belonging to the Myrtaceae family and an important commercial crop in Taiwan. In this study, the protective effects of a diet containing red guava on inflammation and oxidative stress in streptozotocin (STZ)-induced diabetic mice were examined. The experimental group was divided into seven subgroups: normal (N), diabetes mellitus (DM), diabetes + red guava 1% (L), 2% (M), and 5% (H), diabetes + 5% red guava + anti-diabetic rosiglitazone (HR), and diabetes + anti-diabetic rosiglitazone (R). The mice were fed for 8 weeks and sacrificed by decapitation. Compared with the DM group, the experimental groups with diets containing red guava as well as rosiglitazone all showed significant improvements in blood glucose control, insulin resistance, creatinine, blood urea nitrogen, triglycerides, non-esterified fatty acids, cholesterol, c-reactive protein, TNF-α, and IL-10. Furthermore, the expression of inflammatory proteins, such as iNOS and NF-κB, was suppressed via activated PPARγ, and the expression levels of GPx3 and ACO increased. In summary, red guava can significantly suppress inflammatory and oxidative damage caused by diabetes and alleviate diabetic symptoms; thus, it exerts protective effects and has potential applications for the development of a dietary supplement.

## 1. Introduction

Diabetes is a long-course chronic metabolic disease. In modern society, changes in lifestyle and diet are associated with an increasing incidence of diabetes, and this has become a global health issue. By the year 2030, diabetes mellitus is estimated up to about 5% of the world’s population (*i.e.*, 366 million people) [1]. More than 90% of diabetic patients account for type II diabetes. The characteristic of type II diabetes is insulin resistance and glucose intolerance. Therefore, a newer strategy in the treatment of type II diabetes is to reduce insulin resistance in peripheral tissue and control of blood glucose level. The red guava (a red-fleshed guava cultivar of *Psidium guajava* L.) is one of the most important crops belonging to the genus *Psidium* and the Myrtaceae family. It is a tropical or subtropical tree or shrub, and is planted in the western part of Taiwan Island year-round. According to the recorded traditional Chinese folk medicine, guava is commonly used as an antibacterial agent [2] as well as for enteritis [3,4] and diabetes [5,6,7]. The decoction of dried fruits or leaves of guava has anti-hypertensive and anti-diabetic effects; thus, it is a popular herbal beverage in folk medicine. According to previous studies [8], guava contains high levels of dietary fiber, which effectively controls blood glucose. Water-soluble dietary fiber has various additional benefits; it not only adsorbs cholate and reduces blood lipid levels, but it also delays glucose absorption by the intestine, leading to a gradual increase in blood glucose and resulting in reduced insulin secretion. *P. guajava* is used as a traditional medicine in certain cultures. The fruits are known to possess large amounts of vitamins and minerals, and have such high levels of polyphenolic antioxidants. The nature of functional molecules in red guava including anthocyanins, flavonoids, proanthocyanins, sesquiterpenoids and triterpenoids of fruit extract. These antioxidant properties are associated with its phenolic compounds such as protocatechuic acid, ferulic acid, quercetin and guavin B, quercetin, ascorbic acid, gallic acid and caffeic acid [9]. Some investigators suggested that the hypoglycaemic components in guava fruits might involve ursolic acid, oleanolic acid, arjunolic acid and glucuronic acid [10]. Type II diabetes is a complex disease characterized by insulin resistance, leading to pancreatic islet and β-cell dysfunction, hyperglycemia, dyslipidemia, and inflammation [11]. Peroxisome proliferator activated receptor γ (PPARγ) is a ligand activated transcription factor of the nuclear receptor superfamily that controls the expression of a variety of genes involved in fatty acid metabolism, adipogenesis, inflammation and insulin sensitivity. Activation of PPAR lowers plasma triglycerides and elevates plasma HDL cholesterol levels, at the same time increasing insulin sensitivity leading to its anti-diabetic effects [12]. PPAR agonists such as Rosiglitazone are currently being used as potent anti-diabetic agents in conventional medicine [13]. In Taiwanese folk medicine, red guava has long been used to improve diabetes. Some academic studies have confirmed the glucose stabilization and anti-oxidation effects of red guava, but a detailed mechanism has yet to be elucidated, therefore in this study, the effects of dietary red guava on type II diabetic mice were explored.

## 2. Results and Discussion

### 2.1. Results

The influence of dietary red guava on blood glucose in STZ-induced diabetic mice is summarized in Table 1. After inducing diabetes by continuous STZ injection, except in the DM group, the blood glucose levels in mice of the L, M, H, HR, and R groups were not statistically different from those of mice in the N group (*p* > 0.05) after 8 weeks of feeding with experimental diets, suggesting that red guava was beneficial for blood glucose control. The experimental results indicate that the addition of an appropriate amount of red guava in the diet can alleviate hyperglycemia and hyperinsulinemia caused by diabetes.

The influence of red guava in the diet on kidney function in STZ-induced diabetic mice is presented in Table 2. The creatinine levels in mice of the DM group were significantly higher than those in mice of the other groups (*p* < 0.05), and creatine levels in mice of the L, M, and R groups were not significantly different from those in mice of the N group (*p* > 0.05). Therefore, the addition of an appropriate amount of red guava to the diet can improve kidney metabolic abnormalities caused by diabetes. BUN levels were significantly higher in the DM group than that in the N group (*p* < 0.05), and were significantly lower in the L, M and H groups (with red guava added) than in the DM group (*p* < 0.05). The reductions in BUN levels in groups with rosiglitazone treatment were insignificant. Hence, the addition of an appropriate amount of red guava to the diet can improve metabolic abnormalities in kidneys caused by diabetes.

The influence of red guava in the diet on blood lipid levels in STZ-induced diabetic mice is summarized in Table 3. After inducing diabetes by continuous STZ injection, the blood levels of TG, cholesterol, and NEFA were significantly higher in the DM group than in the N group (*p* < 0.05), while blood lipid levels were significantly lower in the L, M, H, HR, and R groups than in the DM group (*p* < 0.05). Hence, the addition of red guava in the diet can improve abnormities in lipid metabolism caused by diabetes.

The influence of red guava in the diet on inflammatory responses in STZ-induced diabetic mice is shown in Table 4. C-RP and TNF-α levels were significantly higher in the DM group than in the N group (*p* < 0.05). The levels of inflammatory mediators were significantly reduced in groups fed red guava-containing diets. Serum IL-10 levels were significantly lower in the DM group than in the N group (*p* < 0.05), while levels of the anti-inflammatory cytokine IL-10 were similar in groups fed red guava-containing diets and the N group. Therefore, the addition of an appropriate amount of red guava to the diet can improve inflammatory responses caused by diabetes.

**Figure 1:**
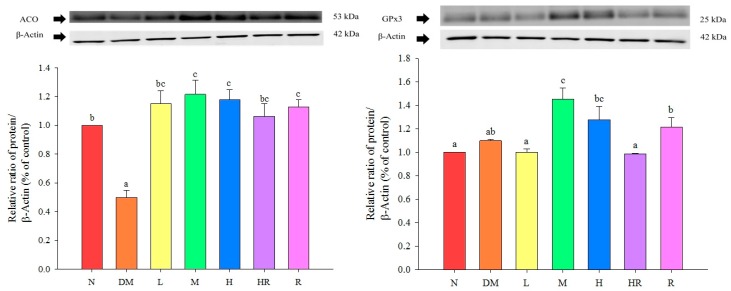
Influence of red guava on ACO and GPx3 protein expression in mouse livers. The treatments were as follows: normal (N), diabetes mellitus (DM), diabetes + red guava 1% (L), 2% (M), and 5% (H), diabetes + 5% red guava + anti-diabetic rosiglitazone (HR), and diabetes + anti-diabetic rosiglitazone (R). Values are expressed as mean ± SD of each group. The lowercase letters above the bars indicate significant differences (*p* < 0.05).

The expression levels of GPx3 were significantly higher in the M and H groups than in the DM and N groups (*p* < 0.05), suggesting that supplementing mouse diets with the proper amount of red guava may increase GPx3 protein expression in the liver; this may improve oxidative damage caused by diabetes. ACO expression levels were significantly lower in the DM group than in the N group (*p* < 0.05), and in all groups fed red guava-containing diets, ACO expression levels were significantly increased in mice livers (*p* < 0.05), which promotes lipid metabolism. These results are shown in Figure 1.

**Table 1:** Influence of red guava on blood glucose, insulin, and insulin resistance.

	N	DM	L	M	H	HR	R
Glucose (mg/dL)	129.06 ± 8.41 ^a^	234.21 ± 62.94 ^b^	174.03 ± 24.97 ^a^	132.69 ± 19.52 ^a^	141.51 ± 24.33 ^a^	141.61 ± 25.18 ^a^	135.83 ± 25.2 ^a^
Insulin (μg/L)	2.13 ± 0.7 ^a^	4.61 ± 1.44 ^b^	2.8 ± 0.46 ^a^	2.33 ± 0.7 ^a^	2.81 ± 0.34 ^a^	2.75 ± 0.69 ^a^	2.36 ± 0.83 ^a^
HOMA-IR	0.68	2.71	1.22	0.98	0.86	0.76	0.79

Results are mean ± SD of each group. Statistically significant differences are indicated by the symbols. Values not sharing letters in the same horizontal column differ significantly from one another by one-way ANOVA and Duncan’s Multiple Range Test (*p* < 0.05) among seven groups. The treatments were as follows: normal (N), diabetes mellitus (DM), diabetes + red guava 1% (L), 2% (M), and 5% (H), diabetes + 5% red guava + anti-diabetic rosiglitazone (HR), and diabetes + anti-diabetic rosiglitazone (R).

**Table 2:** Influence of red guava on creatinine and blood urea nitrogen (BUN).

	N	DM	L	M	H	HR	R
Creatinine (mg/dL)	0.49 ± 0.02 ^b^	0.69 ± 0.04 ^c^	0.46 ± 0.03 ^a,b^	0.43 ± 0.03 ^a,b^	0.38 ± 0.03 ^a^	0.38 ± 0.02 ^a^	0.52 ± 0.03 ^b^
BUN (mg/dL)	14.30 ± 3.35 ^a^	54.70 ± 2.74 ^c^	40.06 ± 2.62 ^b^	37.58 ± 3.13 ^b^	36.09 ± 4.64 ^b^	46.66 ± 1.77 ^b,c^	45.54 ± 4.42 ^b,c^

Results are mean ± SD of each group. Statistically significant differences are indicated by the symbols. Values not sharing letters in the same horizontal column differ significantly from one another by one-way ANOVA and Duncan’s Multiple Range Test (*p* < 0.05) among seven groups. The treatments were as is in Table 1.

**Table 3:** Influence of red guava on triglycerides, cholesterol, and non-esterified free fatty acids.

	N	DM	L	M	H	HR	R
Triglycerides (mmol/L)	1.54 ± 0.03 ^c^	2.45 ± 0.08 ^d^	1.34 ± 0.02 ^a,b^	1.31 ± 0.02 ^a,b^	1.41 ± 0.03 ^a,b,c^	1.28 ± 0.02 ^a^	1.44 ± 0.04 ^b,c^
Cholesterol (mg/dL)	140.74 ± 1.28 ^a^	152.85 ± 2.58 ^b^	139.12 ± 2 ^a^	135.97 ± 0.91 ^a^	137.4 ± 1.7 ^a^	138.25 ± 1.41 ^a^	140.78 ± 2.36 ^a^
NEFA (mmol/L)	1.56 ± 0.03 ^c^	2.05 ± 0.06 ^d^	1.37 ± 0.04 ^ab^	1.43 ± 0.02 ^b,c^	1.47 ± 0.03 ^b,c^	1.26 ± 0.04 ^a^	1.45 ± 0.04 ^b,c^

Results are mean ± SD of each group. Statistically significant differences are indicated by the symbols. Values not sharing letters in the same horizontal column differ significantly from one another by one-way ANOVA and Duncan’s Multiple Range Test (*p* < 0.05) among seven groups. The treatments were as in Table 1.

**Table 4:** Influence of red guava on C-RP, TNF-α, and IL-10.

	N	DM	L	M	H	HR	R
C-RP (ng/mL)	53.77 ± 0.36 ^b^	59.98 ± 1.44 ^c^	54.49 ± 1.91 ^b^	53.87 ± 1.39 ^b^	47.87 ± 1.48 ^a^	55.95 ± 0.68 ^a,b^	57.52 ± 1.11 ^a,b^
TNF-α (pg/mL)	133.86 ± 6.87 ^a^	303.4 ± 36.95 ^c^	264.33 ± 3.18 ^b,c^	232.33 ± 6.96 ^b^	250.67 ± 13.86 ^b,c^	263.33 ± 18.76 ^b,c^	204.67 ± 15.76 ^b^
IL-10 (pg/mL)	243.18 ± 19.6 ^b^	150.27 ± 8.3 ^a^	216.6 ± 19.38 ^b^	214.98 ± 20.29 ^b^	206.64 ± 18.69 ^a,b^	191.31 ± 28.64 ^a,b^	248.68 ± 25.04 ^b^

Results are mean ± SD of each group. Statistically significant differences are indicated by the symbols. Values not sharing letters in the same horizontal column differ significantly from one another by one-way ANOVA and Duncan’s Multiple Range Test (*p* < 0.05) among seven groups. The treatments were as in Table 1.

**Figure 2:**
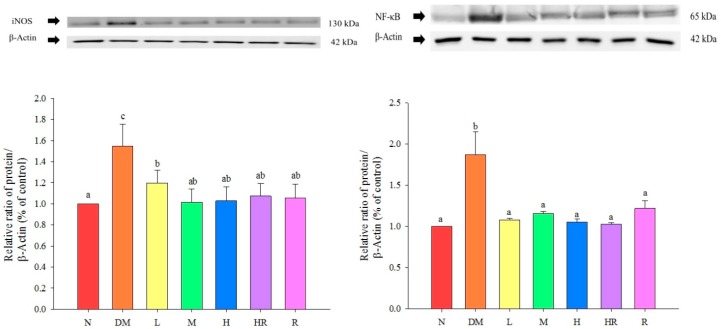
Influence of red guava on iNOS and NF-κB inflammatory protein expression in mouse livers. The treatments were as follows: normal (N), diabetes mellitus (DM), diabetes + red guava 1% (L), 2% (M), and 5% (H), diabetes + 5% red guava + anti-diabetic rosiglitazone (HR), and diabetes + anti-diabetic rosiglitazone (R). Values are expressed as mean ± SD of each group. The lowercase letters above the bars indicate significant differences (*p* < 0.05).

The NF-κB and iNOS expression levels were both significantly higher in the DM group than in the N group (*p* < 0.05), while the levels of these inflammatory proteins in the liver in groups fed red guava-containing diets were significantly lower than they were in the DM group (*p* < 0.05). The results are shown in Figure 2.

**Figure 3:**
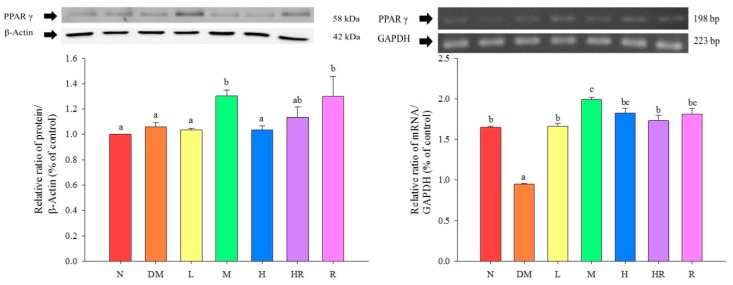
Influence of red guava on PPARγ protein and mRNA expression in the mouse epididymal fat. The treatments were as follows: normal (N), diabetes mellitus (DM), diabetes + red guava 1% (L), 2% (M), and 5% (H), diabetes + 5% red guava + anti-diabetic rosiglitazone (HR), and diabetes + anti-diabetic rosiglitazone (R). Values are expressed as mean ± SD of each group. The lowercase letters above the bars indicate significant differences (*p* < 0.05).

PPARγ expression levels in the epididymal fat were significantly higher in the M, HR, and R groups than in the N group (*p* < 0.05), and were similar among mice in the M, HR, and R groups (*p* > 0.05). The *PPAR*γ mRNA expression levels in the epididymal fat were significantly lower in the DM group than in the other groups (*p* < 0.05), but were similar to the expression levels in the R group, in which mice were fed a red guava-containing diet (*p* > 0.05). These results are shown in Figure 3.

### 2.2. Discussion

In this study, the continuous injection of a small amount of STZ was used to chronically damage pancreatic β cells and reduce insulin secretion; this simulated the reduced insulin action in type II diabetes with insulin resistance. Owing to the insufficient action of insulin, glucose in blood cannot be absorbed and used by cells, leading to an increased blood glucose level, which in turn affects lipid and protein metabolism. However, in order for cells to uptake and utilize blood glucose, the body compensates and forces pancreatic β cells to increase insulin secretion, causing hyperinsulinemia. In these conditions, hyperglycemia and hyperinsulinemia gradually reduce the sensitivity of insulin receptors, and eventually result in insulin resistance and type II diabetes. Insulin resistance, in which the insulin sensitivity of peripheral tissues is reduced, can lead to high levels of glucose and insulin in the blood. This is an important pathological feature and also a diagnostic criterion of type II diabetes. Since insulin resistance is associated with many diseases, early screening for patients with insulin resistance can prevent diabetes. The HOMA-IR (homeostasis model assessment of insulin resistance) index is a stable insulin resistance index; higher values are associated with higher incidences of metabolic syndrome and diabetes, and lower insulin sensitivity [14]. As observed by Qu *et al.*, the HOMA-IR index is typically less than 2.6; populations with HOMA-IR values of 2.6–3.8 have a high risk of insulin resistance, and those with indexes of greater than 3.8 are highly resistant to insulin. As shown in Table 1, this index for mice in the DM group was 2.7, indicating a high risk of insulin resistance, while normal levels were observed for the other groups. We suggest that quercetin, lycopene and ursolic acid in the aqueous extract of red guava fruit promote glucose uptake in liver cells, and contribute to the alleviation of hypoglycemia in diabetes as a consequence [15]. Therefore, we speculated that red guava can reduce insulin resistance, and thereby improve levels of blood glucose and insulin and further alleviate diabetes.

Diabetic nephropathy may occur in both type I and type II diabetes patients, and affects approximately 25%–40% of diabetes patients. Its pathogenesis may be associated with poor blood glucose control, genetic factors, environmental factors, lifestyle, obesity, smoking, *etc.* Diabetic nephropathy is one of the main causes of end-stage renal disease behind type II diabetes [16]. Diabetic nephropathy patients have increased incidence rates of retinopathy, neurodegenerative diseases, peripheral vascular diseases, and coronary artery diseases [17]; thus, early diagnosis and early treatment have become particularly important topics. The kidney functions in the excretion of metabolic waste, and creatinine and BUN in the blood are the most common indicators of kidney function. Creatinine is the production of creatine decomposition in human muscles and is a type of metabolic waste. It is excreted into urine by the kidney and cannot be reabsorbed by kidneys. Upon renal dysfunction and reduced metabolic function, creatinine accumulates in the blood and cannot be excreted from the body, resulting in an elevated blood concentration. Therefore, kidney function can be determined by measuring blood creatinine levels. BUN is the metabolic end product of proteins and amino acids and is normally filtered by glomeruli; 80% can be reabsorbed by kidney tubules and the remainder is discharged via the urine. Therefore, an increased BUN level may indicate kidney dysfunction. Previous studies have found that BUN levels are higher in STZ-induced diabetic mice that in normal mice, which is consistent with the results of this study, and red guava can improve kidney metabolic abnormities caused by diabetes.

Consistent with the results of a previous study [18], our analysis (Table 1) showed that the TG and cholesterol levels were significantly higher in the DM group than in the N group (*p* < 0.05), and this may due to the insufficient production or poor action of VLDL (very low-density lipoprotein) caused by insulin resistance. For hyperinsulinemia caused by diabetes, excessive insulin in the blood can promote the function of HMG-CoA reductase, which in turn further increases the generation of cholesterol. Consistent with previous studies [19], blood NEFA levels were significantly higher in the DM group than in the N group (*p* < 0.05), and this may due to elevated lipolysis caused by insulin resistance. The above results indicate that the addition of an appropriate amount of red guava to diets can promote β-oxidation and the expression of ACO, improving blood lipid metabolic abnormities caused by diabetes.

C-RP is an acute-phase non-glycosylated protein aggregate with a half-life of approximately 19 h. It is highly stable throughout the inflammatory response process. Increased C-RP levels lead to increased incidences of type II diabetes, and are also highly correlated with metabolic syndrome; accordingly, C-RP is an important index of inflammation and atherosclerotic diseases [20,21,22]. According to previous studies, C-RP activates NF-κB in smooth muscle cells [23], suggesting its involvement in the proliferation and activation of smooth muscle cells, and cell accumulation on the inner blood vessel wall, which results in artery injuries. Here, we found that TNF-α levels were significantly higher in the DM group than in the N group. TNF-α is a pro-inflammatory adipokine produced by macrophages that infiltrate the adipose tissue. It can induce insulin resistance via adipokine and fatty acids, and is correlated with obesity and insulin resistance [24]. Therefore, mice in the DM group exhibited inflammatory responses. Based on the results presented in Figure 3, red guava might contain PPARγ activators, suggesting that the addition of a proper amount of red guava to diets may reduce the expression levels of the inflammatory proteins NF-κB and iNOS via activation of PPARγ expression (Figure 2), thus inhibiting diabetes-induced inflammatory responses and increasing GPx3 expression (Figure 1), which further improves oxidative damage in diabetic animals. The results shown in Figure 1 indicate that the addition of an appropriate amount of red guava to diets can accelerate fatty acid decomposition and regulate lipid metabolism via the induction of ACO expression, and can further improve blood lipid metabolic abnormities caused by diabetes. Previous studies have found that 15d-PGJ_2_ (a ligand of PPARγ) increases the mRNA expression of *PPAR*γ [25]. Hence, we speculate that red guava contains PPARγ activators or ligands that bind to the transcription factor PPARγ to inhibit inflammatory responses and improve the outcomes of diabetes.

## 3. Experimental Section

### 3.1. Animal Model

Male BALB/c mice, five-week-old (body weight approximately 15–20 g), were obtained from the National Laboratory Animal Center (National Science Council, Taipei, Taiwan). Mice were housed on a 12 h light/dark cycle. They were first fed a rodent chow diet for one week of adaptation. When their body weights averaged approximately 22 g, we randomly divided them based on their body weights. Mice were fed for a total of eight weeks during the experiment. Feed intake levels during experimentation period were recorded daily, body weight recorded weekly. Each mouse was fed 4 g/day test feed. Excess supply of drinking water was used to allow free intake. After 8 weeks of treatment, mice were sacrificed by decapitation and the blood was removed. After packaging blood and organs obtained, these were cryopreserved in refrigerators at −80 °C for later analysis. Research accorded with internationally accepted principles for laboratory animal use and care, as reviewed and approved by the Institutional Animal Care and Use Committee Guidelines of China Medical University (IACUC, CMU).

### 3.2. Experimental Sample Preparation

**Figure 4:**
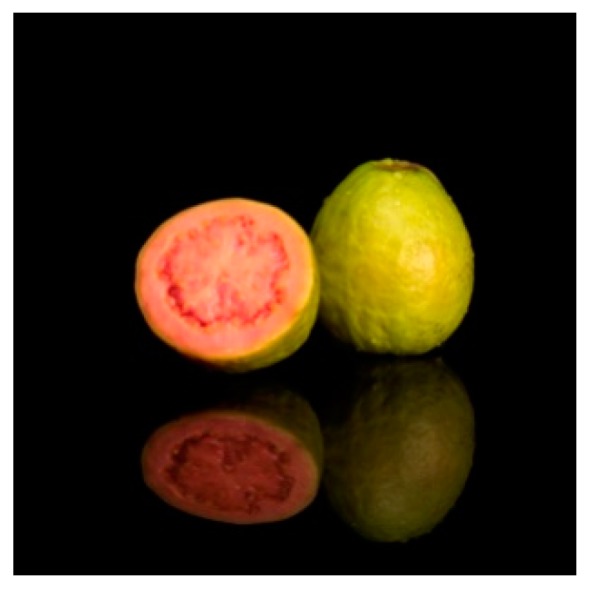
Experimental sample of red guava.

The red guava (red-fleshed guava cultivar of *Psidium guajava* L., Figure 4) obtained in spring 2015 was purchased from farms in Puli Town, Nanton County, Taiwan. A 100 g edible portion of red guava was chopped and mixed with 300 mL sterile distilled water followed by homogenizing in a Waring blender. After filtration through Whatman No. 1 filter paper, the filtrate was further freeze-dried to a fine powder. Sterile water was used to mix freeze-dried red guava powder, the anti-diabetic drug rosiglitazone (Avandia^®^), and the mouse diet at various ratios to prepare the experimental diets for each group. Seven subgroups were defined as follows: normal (N), diabetes mellitus (DM), diabetes + red guava 1% (L), 2% (M), and 5% (H), diabetes + 5% red guava + anti-diabetic rosiglitazone (HR), and diabetes + anti-diabetic rosiglitazone (R).

### 3.3. Establishment of the Animal Model of Diabetes

The method recommended by the Taiwan Ministry of Health and Welfare to induce diabetic animal models was used, with some modifications. Streptozotocin (STZ, 40 mg/kg; Sigma, St. Louis, MO, USA) dissolved in 0.1 M citrate buffer (pH 4.2) was intraperitoneally injected in mice for 5 consecutive days as described previously [26]. Citrate buffer was injected into the control group, instead of STZ. Blood glucose level was monitored on day 10 from the tail vein by using a glucometer (Accu-Chek^®^; Roche, Penzberg, Germany) and blood glucose test strips were used to measure the blood glucose concentration in mice. Successful induction of diabetes was determined according to these results. Specifically, diabetes was successfully induced in a mouse if its blood glucose concentration was greater than 200 mg/dL. Experiments were performed on the next day.

### 3.4. Analysis Biochemical Parameters

Blood creatinine, blood urea nitrogen (BUN), triglycerides (TG), cholesterol, and non-esterified fatty acid (NEFA) were analyzed using commercial analysis kits (Randox Lab, Crumlin, Northland, UK). The serum glucose level (mg/dL) was measured by a glucose HK kit (Sigma). The serum insulin level (μg/L) was measured by a method using a mouse insulin EIA kit (Mercodia AB, Sylveniusgatan 8A, Uppsala, Sweden). Insulin resistance was estimated using the homeostasis model of assessment-insulin resistance (HOMA-IR) formula: (fasting glucose (mmol/L) × fasting insulin (μU/mL)/22.5). The serum inflammatory mediators c-reactive protein (C-RP) was analyzed using clinical test kits (Roche Cobas Mira plus, Berlin, Germany). The serum cytokine levels were determined by commercially available enzyme-linked immunosorbent assay (ELISA) kit (Bio-source International Inc., Camarillo, CA, USA) according to the manufacturer’s instructions. Cytokines TNF-α and IL-10 were determined by a standard curve, concentrations were expressed as pg/mL.

Mouse livers and epididymal fat were homogenized in lysis buffer (0.6% NP-40, 150 mM NaCl, 10 mM HEPES (pH 7.9), 1 mM EDTA, and 0.5 mM PMSF) at 4 °C. Fifty micrograms of protein was subjected to 10% SDS-PAGE (sodium dodecyl sulfate polyacrylamide gel electrophoresis) and transferred to a polyvinylidene fluoride membrane (NEN Life Science, Boston, MA, USA). Immunodetection was performed with the Enhanced Chemiluminescence (ECL) Western Blot Kit (Amersham International, Amersham, UK) using goat anti-mouse PPARγ, nuclear factor-kappaB (NF-κB), inducible nitric oxide synthase (iNOS), Glutathione peroxidase 3 (GPx3), acyl-CoA oxidase (ACO), and β-actin antiserum as primary antibodies (Millipore, Billerica, MA, USA) and biotinylated goat anti-rabbit IgG species-specific whole antibody (Amersham Biosciences, Piscataway, NJ, USA) as the secondary antibody (1:100). Blots were then quantified as relative intensities compared to the control with Kodak Molecular Imaging Software (Version 4.0.5, Eastman Kodak Company, Rochester, NY, USA).

RT-PCR was performed to analyze *PPAR*γ mRNA levels in the epididymal fat mass. cDNA fragments of *GAPDH* (223 bp) and *PPAR*γ (198 bp) were obtained by PCR, and were separated by electrophoresis on a 2% agarose gel containing EtBr. After confirming the size of the PCR products using a DNA marker, the MCID image processing system (Microcomputer Imaging Device-M4 ver 3.0, St. Catherines, ON, Canada) was used for the analysis.

### 3.5. Statistical Analysis

All experimental results are presented as means ± SD of *n* = 12 values. The data were normally distributed, and one-way analyses of variance (ANOVA) and Duncan’s multiple range tests were used to examine differences among independent samples. SPSS 13.0 (SPSS Inc., Chicago, IL, USA) was used for the statistical analysis.

## 4. Conclusions

The results of this study show that the addition of red guava to diets can improve hyperglycemia, hyperinsulinemia, and kidney metabolic abnormities in STZ-induced diabetic mice, and can reduce the expression of the inflammatory proteins iNOS and NF-κB via the activation of *PPAR*γ, resulting in improved inflammatory responses caused by diabetes. Moreover, a red guava-containing diet can increase the expression of GPx3, further improving diabetes-induced oxidative damage, and can increase ACO expression, which improves lipid metabolism and reduces lipid levels in the blood. Red guava can significantly reduce diabetes-induced inflammation and oxidative damage and alleviate diabetic symptoms; thus, it exerts protective effects and a candidate for the development of a dietary supplement.
